# Menstrual Blood as a Potential Source of Endometrial Derived CD3+ T Cells

**DOI:** 10.1371/journal.pone.0028894

**Published:** 2011-12-09

**Authors:** Steffanie Sabbaj, Zdenek Hel, Holly E. Richter, Jiri Mestecky, Paul A. Goepfert

**Affiliations:** 1 Department of Medicine, University of Alabama at Birmingham, Birmingham, Alabama, United States of America; 2 Department of Molecular and Cellular Pathology, University of Alabama at Birmingham, Birmingham, Alabama, United States of America; 3 Department of Obstetrics and Gynecology, University of Alabama at Birmingham, Birmingham, Alabama, United States of America; 4 Department of Microbiology, University of Alabama at Birmingham, Birmingham, Alabama, United States of America; Rush University, United States of America

## Abstract

Studies of T cell-mediated immunity in the human female genital tract have been problematic due to difficulties associated with the collection of mucosal samples. Consequently, most studies rely on biopsies from the lower female genital tract or remnant tissue from hysterectomies. Availability of samples from healthy women is limited, as most studies are carried out in women with underlying pathologies. Menstruation is the cyclical sloughing off of endometrial tissue, and thus it should be a source of endometrial cells without the need for a biopsy. We isolated and phenotyped T cells from menstrual and peripheral blood and from endometrial biopsy-derived tissue from healthy women to determine the types of T cells present in this compartment. Our data demonstrated that T cells isolated from menstrual blood are a heterogeneous population of cells with markers reminiscent of blood and mucosal cells as well as unique phenotypes not represented in either compartment. T cells isolated from menstrual blood expressed increased levels of HLA-DR, αEβ7 and CXCR4 and reduced levels of CD62L relative to peripheral blood. Menstrual blood CD4+ T cells were enriched for cells expressing both CCR7 and CD45RA, markers identifying naïve T cells and were functional as determined by antigen-specific intracellular cytokine production assays. These data may open new avenues of investigation for cell mediated immune studies involving the female reproductive tract without the need for biopsies.

## Introduction

The female reproductive tract is an integral part of the mucosal immune system, and unlike other mucosal compartments, the female reproductive tract is unique because it is required to regulate immune responses that are necessary for protecting the tissue from infectious pathogens while preserving the developing fetus [1], [2]. Thus, this compartment has several distinguishing features that set it apart from other mucosal sites, and allow for unique studies (reviewed in [3]).

Due to difficulties associated with obtaining cells from mucosal tissues, most studies in humans on antigen-specific immune responses at mucosal sites focus on the analysis of humoral responses. However, the female reproductive tract has been shown to possess the same type of cells present in the peripheral blood; in fact lymphoid aggregates present in the cycling endometrium, composed primarily of T cells, have been extensively described [4] [5], [6], [7], [8].

Studies in macaques have indicated the presence of antigen-specific CD8+ T cells in the lower female genital tract [7]. Moreover, studies in humans employing T cells directly isolated from various tissues, including the endometrium or obtained using cytobrush-based sampling from the female reproductive tract have also demonstrated that antigen-specific CD8+ cytotoxic T lymphocyte (CTL) responses are present within this compartment [9], [10]. It has also been demonstrated that these responses are under hormonal control such that CTL responses are lowest during the secretory phase, when both estradiol and progesterone concentrations are highest. Moreover, the highest CTL responses were detected from samples obtained from postmenopausal women where hormone levels are low [10].

The ability to induce antigen-specific T cell responses in the reproductive tract allows for the study of cell-mediated immune responses at mucosal sites in humans. It also provides the impetus for the development of mucosal vaccines capable of protecting against pathogens in these compartments. However, studies using functional T lymphocytes, derived from mucosal tissue, to study antigen-specific responses, T lymphocyte homing and development are challenging due to the difficulty in obtaining genital tissue from healthy women and isolating sufficient numbers of cells.

The uterus is the main immunological organ in the human female genital tract. It contributes most of the IgA for local protection from pathogens [11] and a 90% decrease in the immunoglobulin level from cervical mucus has been shown in hysteterectomized women [12]. Based on this information we sought to determine if the endometrium would also be a source of T cells for the female genital tract that mediated many of the cellular immune responses in this compartment. Menstrual blood, which contains endometrial tissue, is likely to be enriched with these cells limiting the need for biopsies. We isolated and characterized lymphocytes from menstrual blood in order to determine whether this material would be an accurate representation of endometrium-derived T cells from which a plethora of cellular mediated immunological assays could be performed.

## Materials and Methods

### Subjects

Menstruating healthy women (N = 12) and 6 chronically HIV-infected women (for the intracellular cytokine studies only), were recruited from the University of Alabama at Birmingham (UAB) to donate menstrual blood and peripheral blood. Menstrual cups (Diva International, Inc, Kitchener, Ontario) were given to volunteers prior to menstruation and instructions on how to use. Contents of the Diva cup were decanted into 50 ml tubes containing the following: fungizone (0.5 µg/ml), penicillin/streptomycin (100 U/ml) and gentamicin (100 µg/ml) plus ACD (5 mls/tube). Concentrations were determined assuming 25 ml of menstrual blood/tube. Samples were kept at room temperature and brought to the laboratory within 6 hours of collection. In addition seven endometrial tissue samples, derived as anonymous remnant surgical material, were obtained from otherwise healthy women undergoing a hysterectomy. Written informed consent was obtained from all women who participated in this study. The Institutional Review Board of the University of Alabama at Birmingham approved the study.

### Isolation of menstrual blood cells

Menstrual blood was obtained from women on day 1 and/or 2 of the menstrual cycle. Menstrual Blood was diluted 1∶2 in PBS, without Ca^++^/Mg^++^. If the sample had excess mucus, we initially tried to use collagenase and DTT to remove mucous, but addition of these dramatically affected the expression of both CD62L (lymph node homing receptor) and β7 (integrin) on the surface of CD4+ and CD8+ T cells from menstrual and peripheral blood. Following overnight incubation, some of the markers increased, however, the level of expression was still different between the two methods of isolation (data not shown). Therefore, in the studies presented here menstrual blood cells were isolated without the use of either collagenase or DTT. Instead, if sample had excess mucus, it was diluted with PBS, and centrifuged at 400 x g for 10 mins.

Supernatant (SN) was pulled off as to not disturb cells and volume replaced with fresh PBS before layering onto Histopaque (Sigma-Aldrich, St. Louis, MO) and centrifuged at 800 x g for 25 min. RT. Interface containing buffy coat was removed, PBS was added and spun down at 400 x g for 10 minutes twice. If cell pellet contained RBC, it was spun down, supernatant discarded, and pellet re-suspended. To the pellet, 5 mL cold ACK was added for 5 min. Cell lysis was stopped by filling tube with PBS and centrifuged at 400 x g for 10 min. Supernatant was discarded. If pellet still appeared to have RBC, this procedure was repeated up to two more times. Cell pellet was then resuspended in media. If the sample contained too many particulates, the cell suspension was passed through a 70 µM nylon cell strainer (BD Falcon, Franklin Lakes, NJ) to remove residual tissue. PBMC were obtained by standard Histopaque (Sigma-Aldrich, St. Louis, MO) density centrifugation. Cells were counted and used for surface staining and/or intracellular cytokine assays.

### Isolation and purification of lymphocytes from endometrial tissue

Lymphocytes were isolated from tissue segments by dissecting and mincing tissue with surgical scissors to mechanically release the intraepithelial cells and generate a cell suspension. This suspension was passed through a 70 µM nylon cell strainer (BD Falcon, Franklin Lakes, NJ) to remove residual tissue. The remaining cell suspension was washed in RPMI containing 10% AB serum by centrifugation (250 X g for 10 mins). Cells were counted and used for surface marker staining.

#### Surface Marker Staining

Phenotypic characterization of menstrual blood cells (MBC), endometrial tissue and PBMC employed cell-surface markers CD3-Pacific Blue, CD8-PercpCy5.5 or PE, β7-PE, CD29-PE (β1), CD49d-APC (α4), CD62L-FITC, CD103-FITC (αEβ7), HLA-DR-FITC, CD45RO-PeCy7, CD45RA-APC, CD27-PE, CD195-PeCy7 (CCR5), CD197-PECy7 (CCR7), and CD184-APC (CXCR4) (Becton Dickinson, San Jose, CA, USA) and CD4-Alexa750 (eBioscience, San Diego, CA) grouped into five different stains containing 7 different flourochromes in order to maximize information gathered while minimizing the total number of cells needed. For α4β7 analysis, cells were first gated using α4 and either CD4 or CD8 after gating on CD3+CD4+ or CD3+CD8+ cells. Then cells were selected from CD45R0+β7+ gates. In addition, CD19-PeCy7 (B-cells), CD14-PercpCy5.5 (monocytes) and CD56-APC/CD16-PE (NK cells) stains were used to characterize cell composition of the menstrual blood versus PBMC (Becton Dickinson, San Jose, CA, USA). Stained cells were acquired using a BD LSRII flow cytometer (Becton Dickinson [BD], San Jose, CA) and analyzed with FlowJo Version 8.8.6 software (TreeStar, San Carlos, CA). We ran at least 100,000 gated lymphocytes for each stained specimen.

#### Antigens

CMV-pp65 peptide pool (69 18-mers, overlapping by 10 spanning the entire amino acid sequence of the HCMV pp65 protein, New England Peptide, Gardner, MA) (2 µg/ml) or CMV-Lysate (Advance Biotechnologies, Columbia, MD) (2 µg/ml) was used to stimulate T cells. PMA/Ionomycin (25 ng/ml and 500 ng/ml respectively) were used as a positive control.

#### Intracellular cytokine staining

Intracellular cytokine staining was performed as previously described [13]. MBC and PBMC were resuspended in RPMI containing 10% human AB serum and co-stimulatory monoclonal antibodies (anti-CD28 and anti-CD49d; each at 1 µg/ml) were added to each tube along with 50 U/ml of Benzonase (Novagen, Madison, WI). Cells were pulsed with the appropriate antigen followed by the addition of 10 µg/ml Monensin (Golgistop, BD Biosciences, San Jose, CA) and Brefeldin-A (Golgiplug, BD Biosciences, San Jose, CA). Cells were then incubated at 37°C, 5% CO_2_ for 5 hours, and placed at 4°C ON. The following day cells were labeled with a fluorescent LIVE/DEAD fixable Dead cell Stain (Molecular probes, Invitrogen, CA). The surface phenotype of samples was determined by staining with anti-CD3-Pacific Blue, anti-CD4-PercpCy5.5, and anti-CD8-PE antibodies (BD Biosciences, San Jose, CA, USA) for 20 min. at room temperature. Cells were washed in PBS, followed by permeabilization with the Cytofix/cytoperm reagent (BD, San Jose, CA) for 20 min. at room temperature in the dark. Intracellular cytokine staining follows using anti-TNF-α?Pecy7, and anti-IFN-γAlexa700 conjugated antibodies (BD Biosciences, San Jose, CA, USA). At least 100,000 lymphocytes were acquired from each sample using a BD LSR II flow cytometer (Becton Dickinson [BD], San Jose, CA). Data were analyzed using FlowJo Version 8.5 software (TreeStar, San Carlos, CA). Lymphocytes were analyzed based on forward and side scatter profiles after the exclusion of dead cells. Cytokines produced were measured off the CD3+CD4+ or CD3+CD8+ gates relative to the media control values and these gates were applied to all samples with different antigens from the same individual.

#### Statistics

Statistical analyses were performed using the non-parametric Wilcoxon Signed Rank test when linked samples (i.e. PBMC and menstrual blood) from the same individual were compared. When comparisons were made against samples obtained from different individuals (i.e. PBMC or menstrual blood and endometrium), Mann-Whitney U test was used. Analyses were done with Graphpad Prism software 4.0 for Mac. Differences were considered to be significant on the basis of 95% confidence intervals (i.e. p<0.05).

## Results

### Menstrual blood cell composition

**Figure 1:**
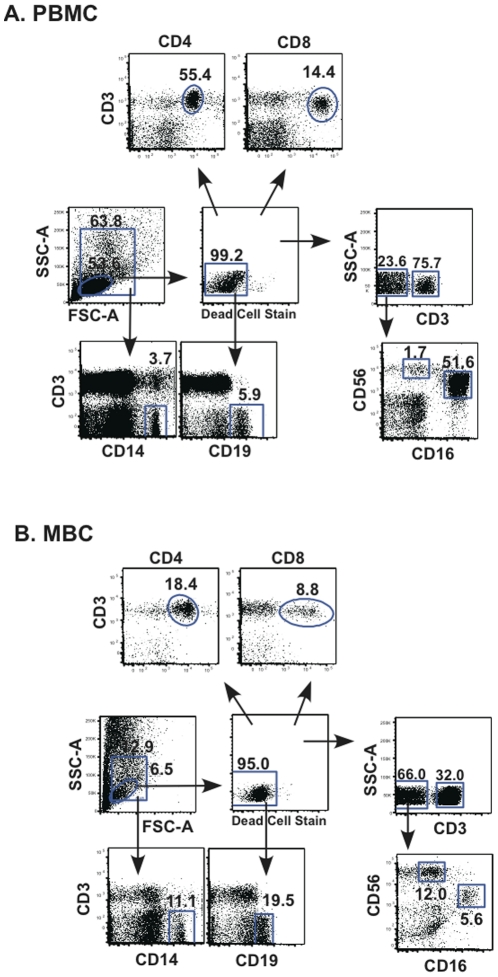
Gating strategy for menstrual and peripheral blood cells. **A.** PBMC and **B.** MBC. Initial gating is performed from the SSC-A vs. FSC-A to determine the total lymphocyte gate (small gate) and the larger gate is used to further gate for monocytes (CD14). From the lymphocytes, live cells are gated, followed by gating B cells (CD19), T cells (CD3), CD4+CD3+ and CD8+CD3+ cells. From the CD3- gate, NK cell phenotypes are selected. CD16-CD56+(immunoregulatory cells) and CD16+CD56+(Cytotoxic cells). Percentages of positive cells for a given marker are indicated above/near gate.

**Figure 2:**
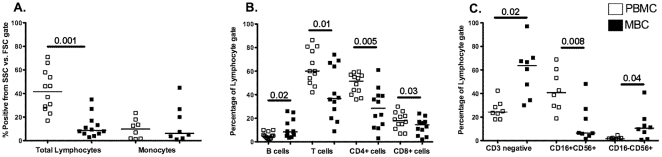
Cell composition of menstrual blood cells (MBCs). The percentage of various cell types present in menstrual blood versus PBMC were resolved by staining with cell type-specific surface markers. **A.** The total lymphocytes, were determined by the percentage of cells within a lymphocyte gate (small cells from FSC vs SSC plots) and monocytes (N = 8), are CD14+ cell from a leuokocyte gate (all cells within the larger FSC vs SSC plot), refer to Figure 1. **B.** Lymphocyte subsets present in menstrual versus peripheral blood. From the live cell gate, B cells were gated on CD19, T cells were gated on CD3 and T cell subsets were further divided into CD4+CD3+ and CD8+CD3+ T cells. **C.** Natural killer cell subsets (N = 8) gated from CD3- cells. CD16+CD56+ (cytotoxic NK cells) and CD16-CD56+ (immunoregulatory NK cells). The median is shown for data from 12 healthy women, except for monocytes and NK cells, where 8 samples were analyzed. Statistically significant differences (p<0.05) were obtained using Wilcoxon Signed Rank test.

Cells obtained from menstrual and peripheral blood were analyzed by staining for antigenic markers of T cells (CD3+CD4+ & CD3+CD8+), B cells (CD3-CD19+), NK cells (CD3-CD56+CD16+ & CD3-CD56+CD16-) and monocytes (CD3-CD14+), the predominant populations present in PBMC (Figure 1). Significant differences between peripheral blood and menstrual blood were seen for all the lymphocyte populations (total, B cells, T cells and NK cells); however, no differences were noted for monocytes (Figure 2A). The immunomodulatory subset of NK cells (CD3-CD16-CD56+) present in menstrual blood, was enriched when compared to peripheral blood (Figure 2C). Conversely, the percentage of cytotoxic NK cells (CD3-CD16+CD56+) was lower in cells obtained from menstrual blood.

### Comparison of surface marker expression

**Figure 3:**
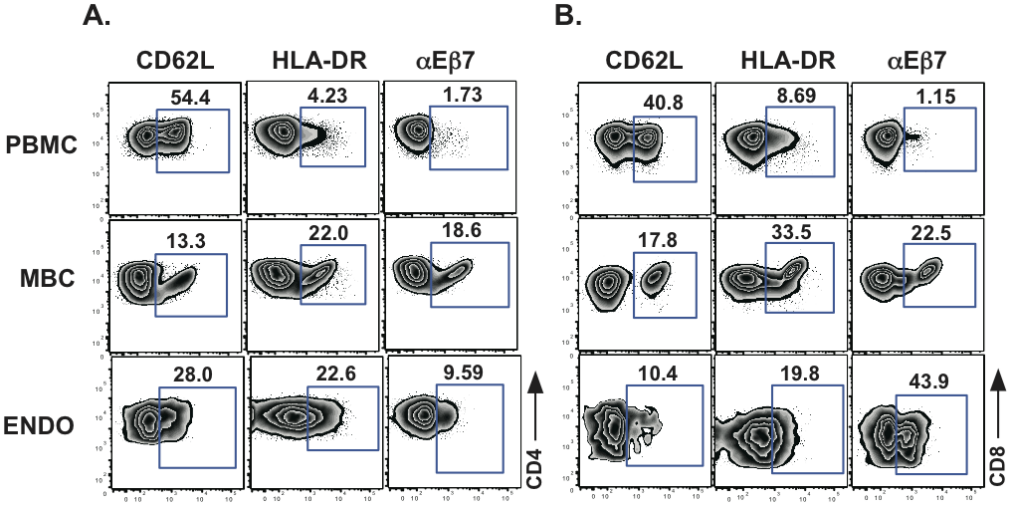
A representative example of surface antigen expression. Cell surface expression of CD62L, HLA-DR and αEβ7 on **A.** CD3+CD4+ and **B.** CD3+CD8+ T cells from PBMC, MBC and endometrial tissue (ENDO) is shown. Percent of positive cells for a given marker are indicated above the gate.

**Figure 4:**
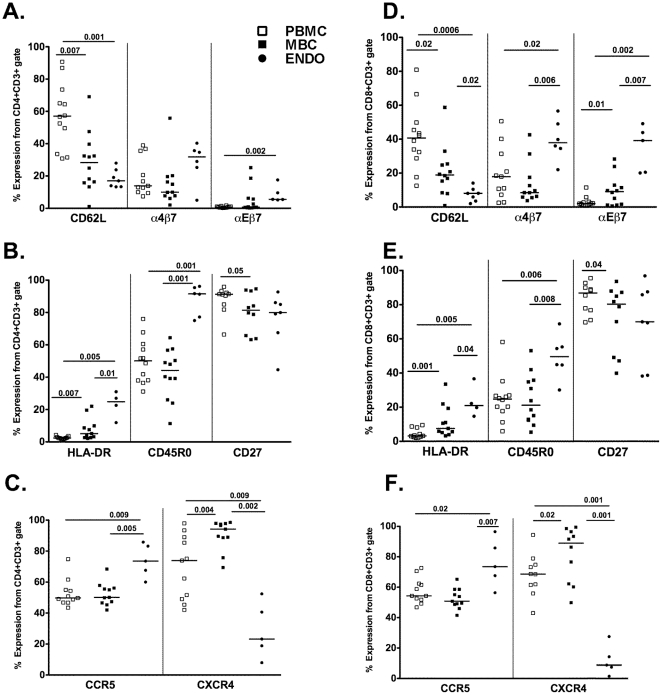
Surface marker expression on PBMC, MBC, and cells from endometrial tissue. Surface markers on CD3+CD4+ cells are shown for panels **A–C** and on CD3+CD8+ on panels **D–F**. The median data from peripheral and menstrual blood is shown for 12 healthy women. Endometrial tissue from 7 women was available for several analyses; however, for some samples, data from only four samples was obtained. Comparisons were made using Wilcoxon Signed Rank test and Mann Whitney U test for paired and unpaired samples, respectively.

To determine the source of lymphocytes present in menstrual blood (i.e. mucosal or systemic), we compared the cell surface markers expressed on cells obtained from menstrual and peripheral blood and endometrial surgical samples. Sample flow plots, gated on CD3+CD4+ and CD3+CD8+ T cells for analysis of one homing and one retention receptor, respectively (CD62L and αEβ7) and an activation marker (HLA-DR), showed differences among the samples (Figure 3). Specifically, the percentages of CD4+ and CD8+ T cells isolated from MBC expressing CD62L (lymph node homing receptor) were significantly lower compared to PBMC but tended to be higher than those isolated from the endometrium (Figure 3, 4A & D). Conversely, the percentage of CD3+CD8+ T cells expressing the mucosal retention receptor αEβ7 (CD103) was higher on MBC when compared to PBMC but lower compared to endometrium-derived cells (Figure 3 and 4D). MBC CD4+ and CD8+ T cells expressing α4β7 was more similar to the percentages observed in PBMC than in endometrium (Figure 4A & D). Other differences noted included an intermediate expression of HLA-DR in both CD4+ and CD8+ T cells, when compared to PBMC and endometrium, CD45R0 expression in menstrual blood cells that mirrored PBMC and CD27 on MBC was more similar to endometrium than PBMC (Figure 4B & E). Interestingly, CXCR4 was elevated on both CD4+ and CD8+ T cells from MBC compared to both PBMC and endometrium (Figure 4C & F). Lastly, no differences in the expression of the homing receptor α4β1, often equated with homing to the genital mucosa, was seen between menstrual blood and PBMC and due to limited sample, α4β1 staining was not done for endometrial tissue samples by flow cytometry (data not shown).

### Memory marker expression among cellular subsets

**Figure 5:**
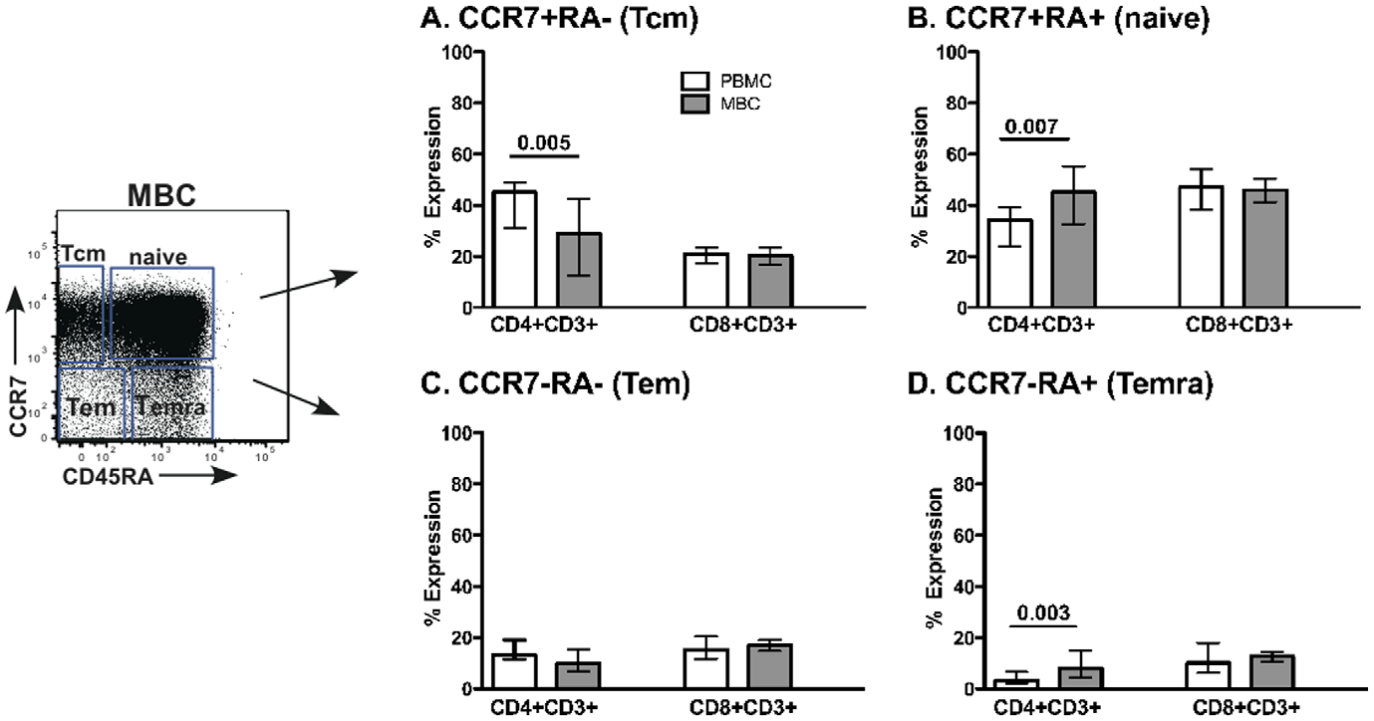
Memory marker expression. CCR7 and CD45RA surface staining was performed on both CD3+CD4+ and CD3+CD8+ T cells from PBMC and MBC. **A–D** represent samples gated on different populations of cells CCR7 and CD45RA expression. The median and interquartile range data is shown for 12 healthy women. Tcm = central memory, Tem = effector memory, and Temra = effector memory RA+. Comparisons were made using Wilcoxon Signed Rank test.

Next, we stained for CD45RA and CCR7 markers, which have been used to distinguish memory cell populations in the peripheral blood [14]. CD4+ T cells from PBMC had a higher percentage of CCR7+CD45RA- (central memory) cells relative to the MBC (Figure 5A). In addition, menstrual blood CD4+ T cells had higher percentage of CCR7+CD45RA+ (naïve) and CCR7-CD45RA+ (terminally differentiated) cells relative to PBMC (Figure 5B&D). No differences were noted between cells from menstrual blood and PBMC for the CD3+CD8+ T cells subsets.

### The frequency of Ag-specific T cell responses

**Figure 6:**
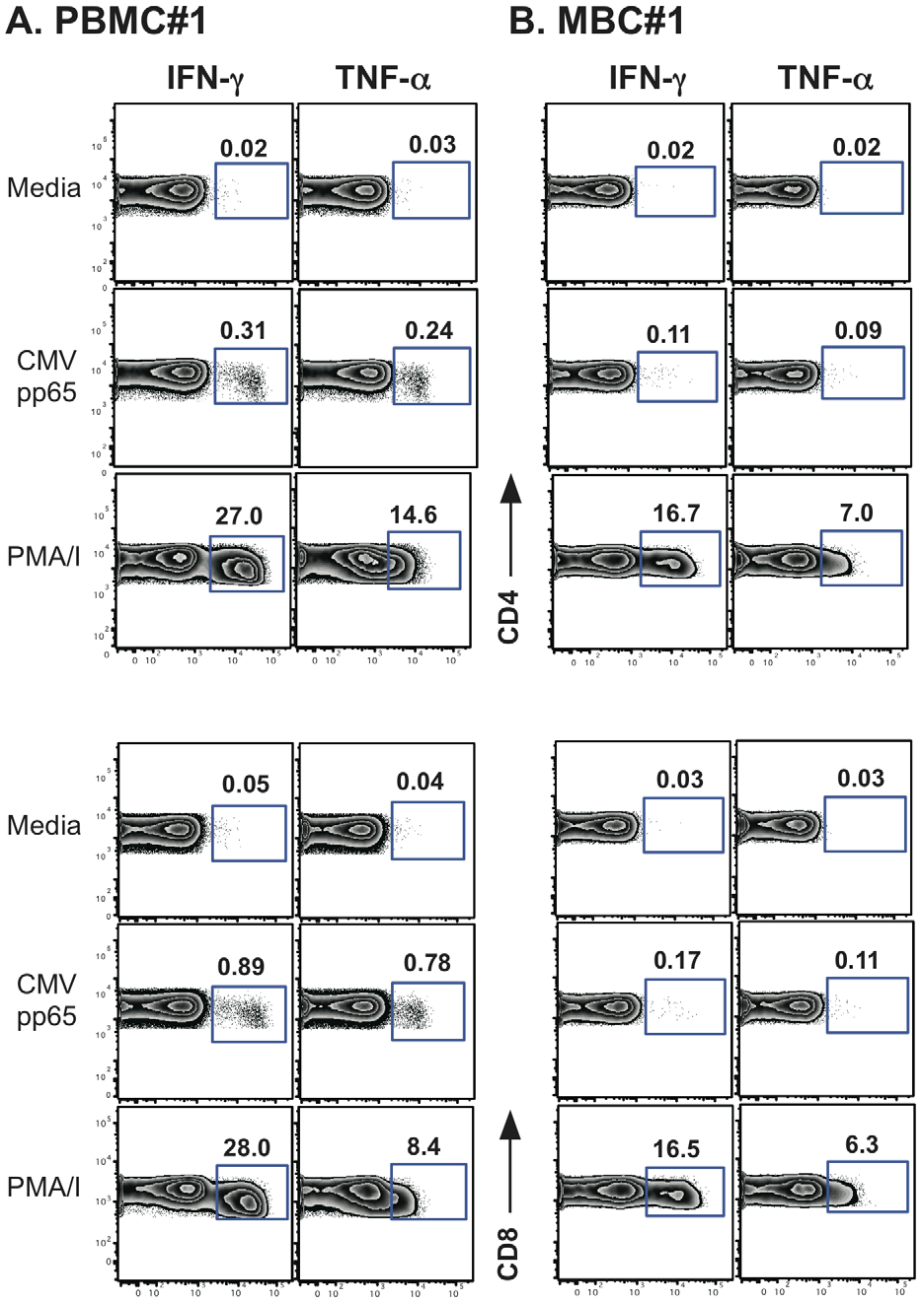
CMV-specific T cells derived from the menstrual blood. **A.** PBMC **B.** MBC from the same healthy woman (#1). Cells were stimulated with overlapping peptides from CMV pp65 and PMA/I as a positive control. Both IFN-γ and TNF-α secretion are shown. Percent of cytokine positive cells (IFN-γ or TNF-α) are shown above the gate.

**Figure 7:**
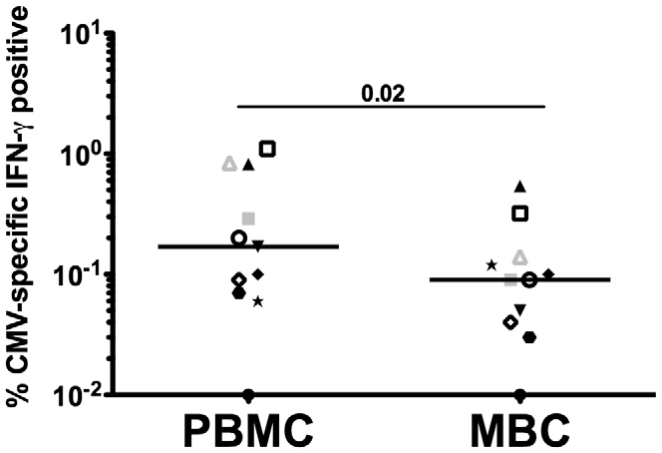
Reduced frequency of CMV-specific T cells derived from the menstrual blood. PBMC and MBC from the same women were stimulated with overlapping peptides from CMV pp65 or CMV lysate and stained for IFN-γ. Individual symbols represent paired samples from the same individual. Open symbols represent CD8 T cell responses, closed symbols represent CD4 T cell responses and gray symbols are responses from the healthy volunteer. Statistical comparisons were made using Wilcoxon Signed Rank test.

To address whether the T cells from the menstrual blood compartment are able to respond to recall antigens, we performed intracellular cytokine staining (ICCS) assays on PBMC from 7 women following stimulation with CMV pp65 overlapping peptides (stimulating both CD4 and CD8 T cells) or CMV lysate (CD4 T cells only). Only a single healthy woman had detectable CMV-specific CD4+ and CD8+ T cell responses obtained from her menstrual blood; however these responses are reduced when compared to PBMC (Figure 6A versus B). Because most of our healthy volunteers did not have responses to CMV measured in their PBMC, we chose to analyze MBC from women chronically infected with HIV since their seroprevalance of CMV should be increased (reviewed in [15]). Indeed, all 6 HIV seropositive women studied had CMV T cell responses detected in their PBMC and menstrual blood. When we compile the data from the one uninfected woman with the data from the 6 HIV-seropositive women, the reduction in CMV-specific T cells from the menstrual blood becomes readily apparent (Figure 7). In fact, in every situation, the frequency of CMV-specific T cells is lower or similar in the menstrual vs. peripheral blood.

## Discussion

We undertook these studies to determine whether menstrual blood cells can be used to analyze T cells present in the endometrium since it has been previously reported that T cells are the predominant lymphocyte from the adaptive immune system in endometrial tissue [4], [5], [6], [8]. However, the proportion of T cell subsets present in menstrual blood seem to mimic the peripheral blood such that both have a higher percentage of CD4+ T cells than CD8+ T cells, unlike the endometrium and other tissue from the female reproductive tract [6], [8], [16], [17], [18]. We also characterized the mononuclear cells present in menstrual blood and showed that many of the T cells present in menstrual blood share similar surface marker expression as those derived from the endometrium. Although the percent monocytes is similar to PBMC, menstrual blood has about 5 fold less total lymphocytes (SSC vs. FCS gate) when compared to PBMC (Median 42% vs 9%, Figure 2A), similar to that reported for other tissue from the female reproductive tract [5]. These differences remained similar when subsets of samples were also analyzed using SSC versus CD45 to gate mononuclear cells (data not shown).

NK cells, a major cell type of the innate immune response, are also present in menstrual blood (Figure 1 and 2C). NK cells can be divided into two main subtypes: cytolytic NK cells (CD16+CD56+) which predominate in the peripheral blood and non-cytolytic regulatory NK cells (CD16-CD56+). In menstrual blood the predominant subset express CD16-CD56+, the phenotype of non-cytolytic regulatory NK cells, consistent with what has been observed using uterine NK cells [19].

Menstrual blood T cells were also shown to be functional and capable of responding to CMV, as measured by IFN-λ secretion using intracellular cytokine staining. Our data show that antigen-specific responses are reduced in T cells obtained from menstrual blood, unlike responses seen in other mucosal compartments such as breast milk and cervical and vaginal tissue [20], [21], [22]. This potential reduction in the number of antigen-specific cells in menstrual blood maybe due to a reduction in the general functional capacity of these cells as they respond to PMA/Ionomycin, but their responsiveness is also reduced when compared to PBMC (Figure 6). This lower level responsiveness may be unique to this compartment, as other studies have also demonstrated decreased function of antigen-specific T cells in cells obtained from endometrial tissue when compared to the peripheral blood [10], [23].

To determine the origin of these cells, we measured the surface expression of homing receptors such as CD62L and the retention receptor αEβ7 and found menstrual blood derived cells expressed an intermediate level of these when compared to the endometrium and PBMC. Endometrial T cells have been shown previously to have an increase expression of HLA-DR relative to PBMC. However, we found that although HLA-DR was elevated compared to PBMC, it was not as elevated as endometrial tissue. Based on this intermediate level of both homing receptors and HLA-DR expression on menstrual blood derived T cells, it appears that these lymphocytes may have originated from both the endometrial and peripheral blood compartments, [16], [24], [25]. Previous data have demonstrated that T cells from tissue sections of endometrium within lymphoid aggregates contained CD8+ T cells where the vast majority (>90%) expressed CD45R0[18]. Our results are inconsistent with these data since we only see this level of CD45R0 expression from CD4+ T cells isolated from endometrial tissue (Figure 4B), and the menstrual blood has CD45R0+ levels analogous to PBMC (Figure 4B&E). However, we cannot rule out the possibility that the cells derived from the endometrium came from older women as compared to those donating menstrual blood and as such these differences can be ascribed to differences in age.

Epithelial cells and lymphocytes obtained from endometrial samples were previously shown to express CCR5 (epithelial cells and lymphocytes) and CXCR4 (epithelial cells only) [26], [27]. These studies readily detected CCR5 on lymphocytes from the ectocervix at all times points during the menstrual cycle. To our knowledge there are no data on the expression of CXCR4 on T cells from the endometrium or upper female genital tract. The expression of CCR5 has been shown to be decreased during the secretory phase of the menstrual cycle where progesterone and estradiol are elevated, yet CXCR4 expression on epithelial cells was not modulated by hormonal fluctuations due to the menstrual cycle [25], [26], [27], [28]. We demonstrated that both chemokine receptors (CCR5 & CXCR4) are readily detected on CD4+ and CD8+ T cells isolated from menstrual blood, however, CXCR4 is significantly elevated when compared to both PBMC and endometrial tissue.

The classification of memory cells and activation state of the cells using CCR7 and CD45RA, respectively is useful when trying to understand whether T cells are antigen experienced without the use of antigen stimulation [14]. Prior data in breast milk demonstrated that cells that have trafficked into the breast are mostly effector memory cells (CCR7-CD45RA-) [29]. This phenotype exemplifies cells that are poised to eliminate pathogens and should not require further differentiation. However, our results (Figure 5) suggest that menstrual blood CD4+ T cells are predominantly CCR7+CD45RA+ (naïve cells) consistent with cells emerging from a lymphoid organ. The endometrium is not a lymphoid organ and thus should not be capable of signaling T cells to differentiate. As such, finding a predominance of CD4+ naïve T cells in this compartment was unexpected. More recently, a consensus was adopted whereby CD45RA, CCR7, CD27 and CD28 markers should be used for the phenotyping of T cell differentiation subsets [30]; however it was previously shown, that CD45RA+CCR7+ cells uniformly co-express CD27 and CD28[31]. We also confirmed in our dataset that naïve cells, as phenotyped by CD45RA+ CCR7+ dual staining, also predominately express CD27 and CD28 (data not shown). The low frequency of CMV-specific responses detected relative to PBMC can also be explained by this predominance of naïve cells (Figure 6A versus 6B and Figure 7).

Recently, hematopoetic stem cells (HCS) and lymphoid progenitors in endometrium expressing CD45RA were described [32]. In the stem cell literature CXCR4 is considered a homing receptor [33], [34] and recently multipotent stem cells have been isolated from menstrual blood [35] and endometrium [36], [37]. These published findings are consistent with the CXCR4 and CD45RA+ data presented in this manuscript suggesting that both HSC and lymphoid progenitors are present in menstrual blood. The monthly regenerative capacity of the endometrium, supports the idea that a subset of menstrual blood CD4 T cells are composed of multipotent cells and has important implications for the establishment of local genital tract lymphocyte repertoires and their regulation and function.

Although obtaining endometrial tissue without the use of a biopsy is an attractive possibility, there are drawbacks for using menstrual blood for the study of endometrial biology. First, the hormonal changes due to the menstrual cycle cannot be followed by using these cells i.e. only one window available to study with these samples. In addition, using menstrual blood is not possible in post-menopausal women or women taking hormonal contraceptives that prevent menstruation. However, although the lymphocytes present in menstrual blood are most likely of mixed origin: blood and endometrium, these cells phenotypically are enriched for cells derived from a mucosal compartment (decreased CD62L and increased αEβ7 expression). Clearly, for certain studies menstrual blood won't replace the use of endometrial biopsies but may be used as a stepping-stone to determine if further studies are warranted before the use of biopsies.
